# Aspergillosis of central nervous system in patients with leukemia and stem cell transplantation: a systematic review of case reports

**DOI:** 10.1186/s12941-021-00452-9

**Published:** 2021-06-15

**Authors:** Aref Shariati, Mojtaba Didehdar, Shahin Rajaeih, Alireza Moradabadi, Mohammad Ghorbani, Vahid Falahati, Zahra Chegini

**Affiliations:** 1 Department of Microbiology, School of Medicine, Iran University of Medical Sciences, Tehran, Iran; 2 Department of Medical Parasitology and Mycology, Arak University of Medical Sciences, Arak, Iran; 3 ENT and Head and Neck Research Center and Department, The Five Senses Health Institute, Firoozgar Hospital, Iran University of Medical Sciences, Tehran, Iran; 4 Department of Medical Laboratory Sciences, Khomein University of Medical Sciences, Khomein, Iran; 5 Division of Vascular and Endovascular Neurosurgery, Firoozgar Hospital, Tehran, Iran; 6 Department of Pediatrics, School of Medicine, Amirkabir Hospital, Arak University of Medical Sciences, Arak, Iran

**Keywords:** Central nervous system aspergillosis, Leukemia, Stem cell transplantation, Fungal infections, Voriconazole

## Abstract

**Background:**

Aspergillosis of Central Nervous System (CNS) is a highly lethal infection in patients with leukemia and Stem Cell Transplantation (SCT).

**Methods:**

Case reports of CNS aspergillosis in patients with leukemia and SCT published between 1990 and August 2020 were gathered using a structured search through PubMed/Medline.

**Results:**

Sixty-seven cases were identified over the searches of the PubMed bibliographic database and then, 59 cases were included in the final analysis. Europe had the largest share of cases at 57.6% (34 reports), followed by Americas and Asia. Affected patients were predominantly males (58.6%) and the mean age of the patients was 36.1 years, while 62.7% of the patients were under the age of 50 years. The most common leukemia types include Acute Lymphoblastic Leukemia (ALL), Chronic Lymphocytic Leukemia (CLL), and Acute Myeloid Leukemia (AML) at 43.4%, 27.4%, and 23.5%, respectively. Furthermore, stem cell transplantation was reported in 11 cases. The overall mortality was 33%; however, the attributable mortality rate of CNS aspergillosis was 24.5%. Altered mental status, hemiparesis, cranial nerve palsies, and seizures were the clearest manifestations of infection and lung involvement reported in 57% of the patients. Histopathologic examination led to the diagnosis of infection in 57% of the patients followed by culture (23.7%), galactomannan assay (8.5%), and molecular method (3.3%). Amphotericin B and voriconazole were the most frequently used drugs for infection treatment. Good results were not obtained in one-third of the patients treated by voriconazole. Finally, neurosurgical intervention was used for 23 patients (39%).

**Conclusion:**

CNS aspergillosis is a rapidly progressive infection in leukemic patients. Thus, these patients should be followed up more carefully. Furthermore, management of induction chemotherapy, use of different diagnostic methods, and use of appropriate antifungal can lead to infection control.

**Supplementary Information:**

The online version contains supplementary material available at 10.1186/s12941-021-00452-9.

## Introduction

*Aspergillus* is a branching septate filamentous fungus that can induce invasive, lethal infections in immune-deficient patients. Among the many species that are identified and recognized, *Aspergillus fumigatus* is by far the most common species Shariati A, Moradabadi A, Chegini Z, Khoshbayan A and Didehdar M [1]. Specifically, *Aspergillus* species can infect respiratory and gastrointestinal tracts and skin, and in patients with immunodeficiency, other forms of the disease can occur. It is quite rare for the Invasive Central Nervous System (CNS) to become subjected to aspergillosis and this phenomenon constitutes about 10–20% of all invasive aspergillosis cases with poor prognosis and significant mortality [2]. *Aspergillus* spp. are common in the environment (soil, dust, plants, and decaying vegetable matter) that are inhaled by breathing normal air, and the lungs have the highest chance of exposure to infection [3]. Thus, the portal of entry for *Aspergillus* usually lies in the respiratory tract and CNS involvement arises as a result of hematogenous spreading from the lung or through direct invasion of the adjacent cranial structure, surgery, contamination of indwelling catheters, and iatrogenic or penetrating trauma [4]. If aspergillosis already invades the paranasal sinuses or palate, it might penetrate the ethmoid sinuses and cribriform plate all the way into the intracranial compartment in which meninges, nerves, lymphatic channels, and blood vessels can become involved [5, 6]. Its extension to the surrounding neural tissues and the vessel wall erosion by hyphal would promote meningitis, hemorrhage, necrosis, vasculitis, and infarction. When *Aspergillus* wears away the arterial wall and attacks the infarcted brain, the sterile infarct will convert to septic infarct and abscess. Thus, meningitis, cerebral blood vessel invasion with secondary infection or hemorrhage, and single or multiple brain abscesses are the highly prevalent forms of CNS aspergillosis reported in patients [7, 8].

Persistent and profound neutropenia is the most significant risk factor in invasive aspergillosis; thus, this infection predominantly occurs in immune-compromised hosts. In this regard, patients with leukemia, recipients of bone marrow transplant, and patients exposed to allogeneic hematopoietic Stem Cell Transplantation (SCT) have a very high chance of developing invasive CNS aspergillosis [1, 9]. For patients with leukemia, especially Acute Lymphoblastic and Myeloid Leukemia (ALL and AML), by receiving intensive cytotoxic chemotherapy and several previous chemotherapy regimens, immunodepression with hypogammaglobinemia inherent to the primary disease and neutropenia caused by infiltration of bone marrow are at increased risk of CNS aspergillosis [10, 11]. Patients with SCT run the high risk of aspergillosis because they are being treated with immunosuppression including high-dose steroids due to the possible spread of the Graft versus Host Disease (GvHD) [12]. Besides, antifungal prophylaxis is recommended for these patients; however, prophylaxis could turn out to be unsuccessful, even with the first-line choices, in about 3–14% of all the patients exposed to invasive fungal infection [4, 13]. Therefore, patients with hematologic malignancies or SCT due to underlying disorders and immunosuppressive therapies have a very high chance of developing CNS aspergillosis and antifungal prophylaxis may not prevent this infection. In addition, given the poor penetration of antifungal agents across the brain-blood barrier, their low concentration in brain tissue and Cerebro-Spinal Fluid (CSF), and their high toxicity, its mortality rate for these patients is quite high [14]. Since little is known about CNS aspergillosis in patients with leukemia or SCT, this systematic review aims to investigate the reported CNS aspergillosis cases in these patients.

## Methods

### Literature search and inclusion criteria

This study carried out a Medline search (via PubMed) from January 1, 1990 to August 30, 2020 with the search keywords obtained from the National Library of Medicine’s Medical Subject Heading (MeSH) terms, abstracts, or titles by using Boolean Operators (and, or): “*Aspergillus*” or “Aspergillosis” and “Leukemia” or “Blood” or “Hematologic” or “Hematological” or “Haematologic” or “Haematological” or “Stem cell transplantation (SCT)” or “Bone marrow transplantation” or “Cytopenia” or “Leukopenia” or “Neutropenia” and “Cerebral” or “Cranial” or “Central Nervous System (CNS)” or “Brain” or “Meningitis”. Article references were reviewed and cross checked at length for any possible additional cases that might have been missed out or overlooked throughout the initial search. It is noteworthy to mention that non-English language studies were excluded. The review protocol used in this study centers on the paper of Hickey et al. and our recent article [15, 16].

### Inclusion criteria

All reports of CNS aspergillosis in patients with leukemia or SCT, full-text or abstract-only studies in English, and research works online in Medline (via PubMed) (from 1990 until August, 2020) were eligible for study inclusion and they were carefully screened by both authors (AS and AM).

### Exclusion criteria

**Fig. 1:**
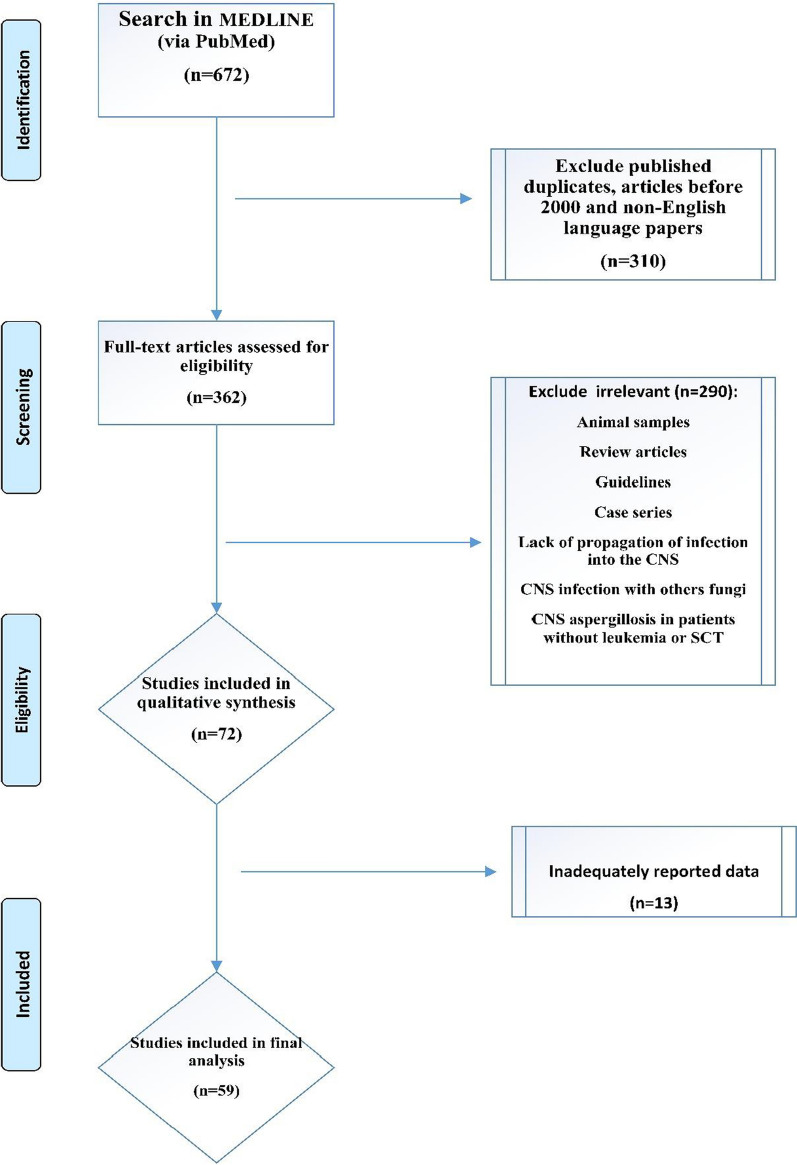
Flow chart of publication selection and their inclusion in the systematic review

The exclusion criteria comprised CNS infections with other fungi, review articles (either systematic or meta-analysis), non-human study, guidelines, CNS aspergillosis in patients without leukemia or SCT, non-propagation of infection into the CNS, and inadequate reported data (Fig. 1).

### Study selection and data extraction

As mentioned earlier, the two researchers (AS and AM) screened the articles and in case of any discrepancy, both researchers were obligated to scan the paper or conference abstract to ensure its eligibility for the review. Individual case reports were considered so as to collect data about the epidemiology, clinical manifestations, treatment, and diagnosis of CNS aspergillosis in patients with leukemia and SCT. Finally, the following features of each pertinent article were extracted and recorded by using Excel software (Microsoft, Redmond, WA, USA): country, year of publication, age, sex, causative fungal pathogen, leukemia, clinical presentation, treatment, surgery, diagnostic methods, and outcome.

### Quality assessment

A critical appraisal checklist was employed for the case reports provided by the Joanna Briggs Institute (JBI) to carry out a quality assessment of the studies [17].

## Results

### Epidemiology

Sixty-seven cases were detected using searches through the PubMed bibliographic database as case reports. Five additional cases were identified and the above-cited references were screened further. Then, thirteen cases were excluded because the leukemia cases of CNS aspergillosis were not analyzed. Finally, 59 patients with leukemia or SCT and CNS aspergillosis were included in the final analysis based on the study criteria (Fig. 1). These cases of individuals were published from USA (14 reports), France (eight reports), Turkey (seven reports), Japan (six cases), Germany and Italy (four each), United Kingdom (three reports), Australia (two reports), Austria, Belgium, China, Czech, Greece, India, Iran, Netherlands, Portugal, Spain, and Sweden (one each). Thus, Europe had the largest share of cases at 57.6% (34 reports), followed by the continents including Americas, Asia, and Australia at 23.7% (14 reports), 15.3% (9 reports), and 3.4% (2 reports), respectively. No case from Africa was found. Our analysis also showed that 58.6% of the patients were male and the rest were female. The mean age of the patients was 36.1 years (ranged from 1.5–90 years), 62.7% of whom were under the age of 50 years (Table 1). Notably, 38.9% of these cases were 18 or younger.

**Table 1:** Clinical manifestations, diagnosis, epidemiological and therapeutic features of central nervous system aspergillosis in patients with leukemia and stem cell transplantation (PubMed reported cases until August 2020)

Country, year of publication and references	Sex/age	Pathogen	Leukemia	Clinical presentation and	Treatment	Surgery	Diagnosis	Outcome
Australia, 1995 [45]	M/2	*A fumigatus*	ALL	Cough, rhinorrhea, fever, myoclonic jerking of the left side of his body	AMB. LAMB, itraconazole	A right frontal craniotomy, a frontal lobectomy, and a left parietal craniotomy	HE, culture	Alive
Australia, 2019 [25]	66/M	*A. felis* complex	CLL	Confusion and expressive dysphasia	Voriconazole, posaconazole	Craniotomy and excision of the left parietotemporal lesion	HE, culture and PCR	Alive
Austria, 1999 [46]	59/M	*A. fumigatus*	ALL	Headache and aphasia	AMB, LAMB, itraconazole	NR	HE, culture	Died
Belgium, 1999 [7]	11/F	NR	ALL	Frontal headache, pyrexia, lethargy and left-sided hemiparesis without meningeal signs	AMB	The entire abscess was removed surgically	HE, negative CSF culture	Died
China, 2015 [18]	53/M	NR	APL	Confusion, left lower extremity movement disorder, and dyspnea	Itraconazole, caspofungin	NR	Culture of sputum	Alive
Czech Republic, 2005 [10]	16/F	NR	B-ALL	Painful severe peripheral neuropathy, gross, frontal behavior, followed by qualitative and quantitative changes in her consciousness	AMB	Open neurosurgical removal	HE	Alive
France,2018 [37]	65/M	*A. fumigatus*	CLL	Headedness, balance disorder, and right hemiparesis (90 days after starting treatment with ibrutinib)	Voriconazole, LAMB and reduction of the ibrutinib dose	NR	Culture	Alive
France, 2001 [47]	30/F	*A. fumigatus*	CML	Fever and right sided pleuritic chest pain	AMB, Flucytosine	NR	Culture	Died
France, 2003 [33]	57/M	NR	AML and PSCT	Fever then the patient became withdrawn and confused	AMB, caspofungin/voriconazole	NA (Due to the bad condition of the patient)	GM assay	Alive
France, 2018 [37]	75/M	*A. fumigatus*	CLL	Fever, visual impairment and ataxia, generalized seizure	Voriconazole, Prednisone	NR	HE, PCR, negative CSF culture	Alive
France, 2019 [26]	39/F	*A. fumigatus*	CLL	Headache, seizures, and fever	LAMB, voriconazole, caspofungin and	Radical surgery	HE of surgical samples and culture	Not reported
France, 2019 [48]	69/M	*A. fumigatus*	CLL	Neurological signs	Voriconazole, ibrutinib was stopped	NR	Culture of BAL	Alive
France, 2019 [32]	52/F	*A. fumigatus*	CLL	Fever, confusion, behavior disorders and aggression	LAMB, voriconazole, isavuconazole, ibrutinib	NR	Culture of brain biopsy	Alive
France, 2020 [31]	69/M	*A. fumigatus*	CLL	Left miosis and a balance disorder	LAMB, voriconazole, isavuconazole, ibrutinib was discontinued	NR	Culture of BAL	Alive
Germany, 1997 [49]	F/62	NR	AML	Fever, Speech was slow and non-fluent, the patient was disoriented, alexic and had homonymous hemianopsia to the right	LAMB and itraconazole	Stereotactic fine-needle aspiration	HE	Alive
Germany, 1997 [23]	18/m	NR	ALL	Fever, sudden onset of pleural chest pain and a pulmonary infiltrate (30 days)	AMB, LAMB, itraconazole, voriconazole	NR	HE, negative culture	Died
Germany, 2017 [24]	22 month/M	*A. fumigatus*	T-ALL	Fever and lymphadenitis, left sided hemiparesis	LAMB, caspofungin, Voriconazole	Neurosurgery with postoperative external ventricular drains	HE, negative culture	Alive
Germany, 2017 [50]	52/M	NR	T-LGL	Acute strong nausea, vomiting, fever, relapsing focal seizures of his right arm, paresthesia and motoric weakness	Voriconazole, LAMB	NR	HE, negative culture and PCR	Alive
Greece, 2013 [51]	32/M	*A. fumigatus*	AML	Weight‑loss, frequent rhinorrhagia, gum swelling, cephalalgia, fatigue, fever, and somnolence, motor nor sensory deficits	AMB	NR	HE, culture	Died
India, 2012 [14]	14/M	NR	B-ALL	Unconsciousness with anisocoria, papilledema and a fixed right-sided gaze	AMB	NR	GM assay	Died
Iran, 2020 [4]	18 month/M	*A. fumigatus/ A. niger*	B cell-ALL	Fever, abnormal focal movement in his right upper limb, loss of consciousness, and seizure	LAMB, voriconazole, Caspofungin	External drainage and hemorrhage drained	HE, culture, PCR	Died
Italy, 2003 [11]	53/F	*A. flavus*	CLL	Left hemiparesis	AMB, voriconazole	NR	Culture	Died
Italy, 2011 [42]	65/M	*A. fumigatus*	CLL,	Fever, headache, pontocerebellar angle syndrome	Caspofungin	A left suboccipital retro sigmoidal craniotomy	HE	Alive
Italy, 2018 [40]	57/	*A. fumigatus*	CLL	Fever and dyspnea	Voriconazole, ibrutinib was stopped	NR	HE	Alive
Italy, 2019 [3]	3/F	NR	B-ALL	Fever and pancytopenia, seizure	Voriconazole then LAMB and isavuconazole	NR	MRI,CT and GM assay	Alive
Japan, 2004 [21]	15/F	NR	PSCT	High fever, headache and weakness in the left hand and leg, and a neurologic examination revealed hemiparesis (1 month after transplantation)	Fluconazole	NR	Panfungal PCR	Alive
Japan, 2007 [20]	33/M	NR	AML	A sudden fever, severe headache, decreased consciousness level and a stiff neck	AMB, voriconazole	NR	GM assay, PCR	Alive
Japan, 2008 [52]	18/M	NR	PSCT	Tachypnea and hypoxemic 30 days after BMT and 1 day after SCT	Micafungin, AMB, flucytosine	NR	PCR, culture, autopsy	Died
Japan, 2008 [44]	15/M	NR	BMT	Left ear pain, and left facial nerve palsy 21 days after BMT, declining level of consciousness as well as right hemiplegia on day 196 after BMT	AMB, itraconazole, micafungin and Voriconazole	NA but catheter coil embolization performed	HE	Alive
Japan, 2020 [19]	90/M	NR	CLL	Headache, fever with altered mental status	Voriconazole	NR	GM assay	Alive
Japan, 2020 [29]	15/M	NR	AML	Dry cough, high fever, right-sided weakness and impaired consciousness	Voriconazole, LAMB and itraconazole	NR	GM assay	Died
Netherlands, 2008 [34]	16/F	*A. fumigatus*	B-ALL	Fever and febrile neutropenia	Voriconazole, caspofungin	NR	Culture	Alive
Portugal, 2005 [12]	52/F	NR	HSCT	Fever, declining level of consciousness 11 month after transplantation	LAMB, fluconazole	Surgery	HE	Died
Spain, 1997 [53]	43/M	NR	ALL	Fever, dyspnea and pleuritic chest pain, then lost consciousness	AMB	NR	HE	Died
Sweden, 2012 [54]	59/M	*A. fumigatus*	B-ALL,	Fever, dysphasia and weakness of his right hand, seizures (12 days)	Voriconazole, LAMB, caspofungin, posaconazole	NR	Culture of BAL and GM assay	Alive
Turkey, 1997 [55]	18/F	*A. fumigatus*	BMT	Fever, headache and hemiparesis	LAMB, itraconazole	Aspiration	Culture	Alive
Turkey, 2002 [43]	45/F	*A. fumigatus*	PSCT	Ataxia and gait disturbance	AMB, itraconazole	Surgical removal of the cerebellar abscess with suboccipital craniectomy	HE	Alive
Turkey, 2012 [2]	4/F	*A. niger*	B-ALL	Right focal seizures	LAMB, voriconazole	Not feasible because of the critical localizations of the lesion	HE and culture	Alive
Turkey, 2013 [35]	4/F	*A. fumigatus*	ALL	Fever, sore throat and headache and focal seizures in the left arm	AMB and voriconazole	NR	HE, culture, PCR of tissue sample	Alive
Turkey, 2018 [56]	21/M	NR	SCT	Vocal cord paralysis and swallowing difficulty (one week after transplantation)	AMB	Abscesses in the brain stem and occipital lobes were totally removed	HE	Died
Turkey, 2018 [56]	18/M	NR	ALL	Clouding of consciousness and tendency toward sleepiness	AMB	Lesion was surgically excised	HE	Alive
Turkey, 2018 [56]	45/F	NR	PBST	Ataxia (two months after transplantation)	AMB	Cerebellar abscess was surgically removed	HE	Alive
UK, 2000 [57]	2/F	*A. fumigatus*	B-ALL	Seizure	AMB and Flucytosine	NR	HE, culture	Died
UK, 2006 [58]	34/M	*A. fumigatus*	AML	Fever, pleuritic pain, left upper motor neuron facial nerve palsy	AMB, LAM, Voriconazole	NR	Culture	Died
UK, 2015 [36]	3/M	*A. nidulans*	B-All	Fever, leg pain, spontaneous bruising, and a petechial rash, right hemiparesis and aphasia	Voriconazole, LAMB then caspofungin and G-CSF	NR	HE, PCR from CSF	Alive
USA, 1991 [5]	M/46	NR	APL	Hemoptysis, lower lobe pneumonic infiltrate, left peripheral facial palsy	AMB	Resection of the inferior temporal lobe abscesses	HE, culture	Alive
USA, 1998 [6]	15/f	NR	ALL	Right lower quadrant pain, fever and neutropenia, and right leg weakness, flaccid paraplegia bilaterally with a sensory level at T8	AMB	NR	Postmortem examination	Died
USA, 2005 [41]	6/F	NR	AML	The patient febrile and showed signs of septicemia	LAMB, voriconazole	Lobectomy and Image-guided stereotactic resection of the lesion	HE	Died
USA, 2005 [41]	6/M	NR	AML	Mouth sores, neurological deficits	LAMB, voriconazole	Lobectomy and Image-guided stereotactic resection of the lesion	HE	Alive
USA, 2005 [41]	16/F	NR	ALL	Seizure	Voriconazole	Image-guided stereotactic resection of the lesion	HE	Alive
USA, 2012 [22]	55/F	*A. terreus*	AML	Mild headache, nonproductive cough and fever, lethargic and Confused	Voriconazole, intrathecal AMB	Stereotactic aspiration and drainage	GMS stains, culture	Died
USA, 2014 [27]	24/F	NR	AML	Fever, chills, diarrhea, and malaise	LAMB, voriconazole, caspofungin	Neurosurgical evacuation of brain abscesses	HE	Alive (Multiple brain abscesses)
USA, 2014 [59]	32/F	NR	T-ALL	Septic shock, febrile neutropenia, acute hypoxic respiratory failure, a right facial droop, severe aphasia, with right upper and right lower extremity paresis	Voriconazole	NR	GM assay	Alive
USA, 2014 [28]	19/F	NR	ALL	Extremity weakness and fevers	Voriconazole	T11–L1 laminectomy	Culture, GM assay	Died
USA, 2018 [38]	48/M	*A. fumigatus*	B cell-ALL	Left eye progressive vision loss, tearing, and redness then declining mental status	Voriconazole and AMB	NR	HE, culture	Alive
USA, 2018 [39]	76/M	*A. fumigatus*	CLL/SLL	Encephalopathy and weakness in his left arm, frontal headache and changes in vision in left eye	voriconazole and micafungin, ibrutinib discontinuation	NR	Culture	Alive
USA, 2019 [60]	62/m	*A. fumigatus*	CLL	Fevers, aphasia, confusion and profound expressive aphasia	Voriconazole, micafungin, isavuconazole Ibrutinib was discontinued	NR	HE, culture	Alive
USA, 2019 [30]	79/M	*A. fumigatus*	B-cell lymphoma/leukemia	Confusion, anorexia, and failure to thrive	Voriconazole, caspofungin	NR	HE, culture	NR
USA, 2020 [61]	74/M	NR	CLL	Fluctuating, slowly progressive imbalance, unsteady gait, left occipital headache and intermittent confusion	Voriconazole	Emergent craniotomy	HE, culture	Alive

*NR* not reported, *NA* not applicable, *HE* Histopathologic Examination, *MB* Amphotericin B, *LAMB* Liposomal Amphotericin B, *CLL* Chronic lymphocytic leukemia, *ALL* Acute lymphoblastic leukemia, *AML* acute myeloid leukemia, *CML* Chronic myelogenous leukemia, *BMT* Bone Marrow Transplantation, *PSCT* peripheral stem cell transplantation, *GMS* Grocott-Gomori's Methenamine Silver, *GM* galactomannan, *APL* Acute promyelocytic leukemia, *T-ALL* acute T-lymphoblastic leukemia, *T-LGL* T-cell large granular lymphocytic leukemia, *SLL* small lymphocytic lymphoma

**Fig. 2:**
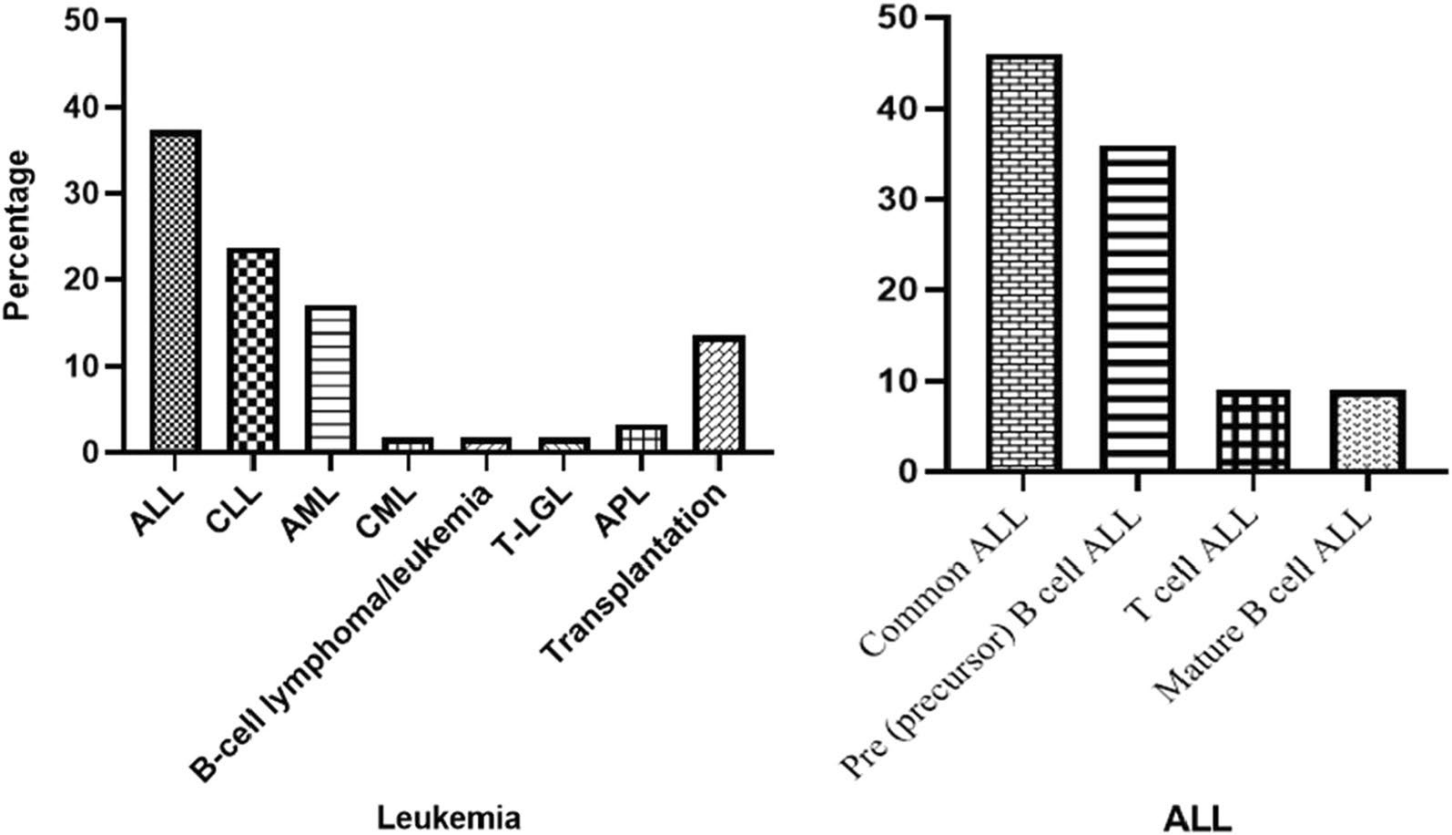
Different leukemia in patient with CNS aspergillosis ((PubMed reported cases until August 2020). *CLL* Chronic lymphocytic leukemia. *ALL* Acute lymphoblastic leukemia. *AML* acute myeloid leukemia. *CML* Chronic myelogenous leukemia. *APL* Acute promyelocytic leukemia. T-ALL: acute T-lymphoblastic leukemia. T-LGL: T-cell large granular lymphocytic leukemia.

The most common type of leukemia associated with CNS aspergillosis was ALL at 43.4% followed by Chronic Lymphocytic Leukemia (CLL) and AML (two case with Acute promyelocytic leukemia (APML, APL)) at 27.4% and 23.5%, respectively (Fig. 2). Different transplantations were reported in 11 patients (18.6%) that include allogeneic bone marrow and SCT for aplastic anemia in four patients, allogeneic bone marrow transplantation for patient with CML, allogeneic SCT for two patients with multiple myeloma and AML, autologous SCT for breast cancer (two patients) and osteosarcoma and finally, cord blood transplantation for patients with AML (Table 1).

Our analysis showed that the total mortality rate in this cohort of published leukemia and SCT cases of CNS aspergillosis was 33% (19 cases) (two studies were omitted from final analysis due to the patients’ failure to adhere to proper follow-up). Among the dead patients, 14 cases (24.5%) died of infection, while CNS aspergillosis was controlled in the other five patients and they, instead, died of hematologic disease progression (ALL and AML (each two cases) and CLL). The highest mortality rate was observed for patients with ALL (47.3%), followed by AML (26.4%) and SCT for Aplastic anemia and multiple myeloma (15.7%). Notably, two patients with CLL and CML also died. Among the patients who died, 52.7% were men and the rest were women. The mean age of the dead was 26.2 years (ranging from 1.5–59 years).

Only 29 studies (49%) performed species-level identification and found that *A. fumigatus* was the most common pathogen isolated from patients with 23 reports. *A. felis, A. flavus, A. niger, A. nidulans*, and *A. terreus* were the other pathogens isolated from patients. Noteworthy, *A. fumigatus* and *A. niger* were also isolated from the patients with pulmonary and cerebral aspergillosis and the mixed breakthrough invasive fungal infections caused the death of patients (Table 1).

### Clinical manifestations

The most common presented manifestations were fever (59.3%), altered mental status (confusion, lethargy or loss of consciousness) (45.7%), headache (23.7%), hemiparesis (17%), facial palsy (8.4%), generalized seizure (10%), ataxia (5%), and focal seizure (6.7%). Imaging modalities showed lung involvement in 57% of the patients, but chest pain was reported only in 8.4% of the cases. In this section, we have divided the duration of onset of clinical symptoms in patients after treatment into three categories: patients undergoing chemotherapy, ibrutinib, and transplant patients. The duration of onset of symptoms in patients who had undergone chemotherapy was determined in 22 cases and the average duration was 27.8 days (mean ± SD = 27.8 ± 16.8 days, ranging from 4 to 80 days). The time of onset of symptoms in patients under ibrutinib therapy was reported in 10 cases with a mean duration of 5.9 months (mean ± SD = 5.8 ± 6 month, ranging from 15 days to 18 month). Finally, in transplant patients, symptoms developed over 4.9 months on average (mean ± SD = 4.8 ± 6.5 month, ranging from 1 week to 22 months) after transplantation. As mentioned, in more than half of the patients, pulmonary infection was also reported and the duration of the onset of CNS aspergillosis symptoms following the detection of lung involvement was reported in 21 cases, which was 18.5 days on average (mean ± SD = 18.5 ± 14.6 days, ranging from 2 days to 2 months). Noteworthy, based on various clinical and laboratory findings, brain lesions (abscess or multiple abscesses), cerebral blood vessel invasion with secondary infection or hemorrhage, and meningitis were the most common CNS disorders caused by *Aspergillus* species in patients at 86.4%, 18.6%, and 3.3%, respectively.

### Diagnoses

The diagnosis was performed by histopathologic examination of different specimens in 23 of 59 (39%) instances. Furthermore, results obtained from culture and histopathologic examination led to the diagnosis of the infection in 11 (18.7%) patients. In three other cases, CNS aspergillosis was diagnosed post-mortem (5%) and culture results confirmed infection in three (5%) other patients. The samples used for diagnosis were obtained by craniotomy, stereotactic and burr hole biopsy, and laminectomy. In light of the formation of abscess capsule, transcranial puncture was not performed on one of the patients [18].

The results of culturing the CNS tissue samples were reported in 19 patients, of whom only three patients (15.7%) were tested negative. On the other hand, the results of CSF culturing were stated in 15 cases and all of them were negative and only one positive case was reported in one patient with thoracic spinal cord intramedullary *Aspergillus* invasion. Our analysis also showed positive cultures from initial vitrectomy, subcutaneous nodules, and surgical joint biopsy. Finally, it should be noted that all blood cultures were negative.

With respect to laboratory tests, *Aspergillus* galactomannan (GM) antigen assay was another diagnostic method that detected CNS aspergillosis in five (8.5%) other patients (lower limit for a positive result 0.5 ng/mL). In one of these patients, negative CSF culture and polymerase chain reaction (PCR) disrupted the diagnosis process; however, serum and CSF samples tested positive for *Aspergillus* GM (more than 5.0), which led to correct identification of the cause of the infection and choice of an appropriate treatment [19]. *Aspergillus* GM results were stated in 28 cases. In this regard, the most positive results of this test were reported for serum (18 (72%) positive, 7 negative), bronchoalveolar lavage (BAL) (2 (50%) positive, 2 negative), and CSF (7 (43%) positive, 9 negative) samples, respectively. *Aspergillus* GM index was reported in 12 cases with an average of 3 ng/mL (Additional file 1: Table S3).

**Fig. 3:**
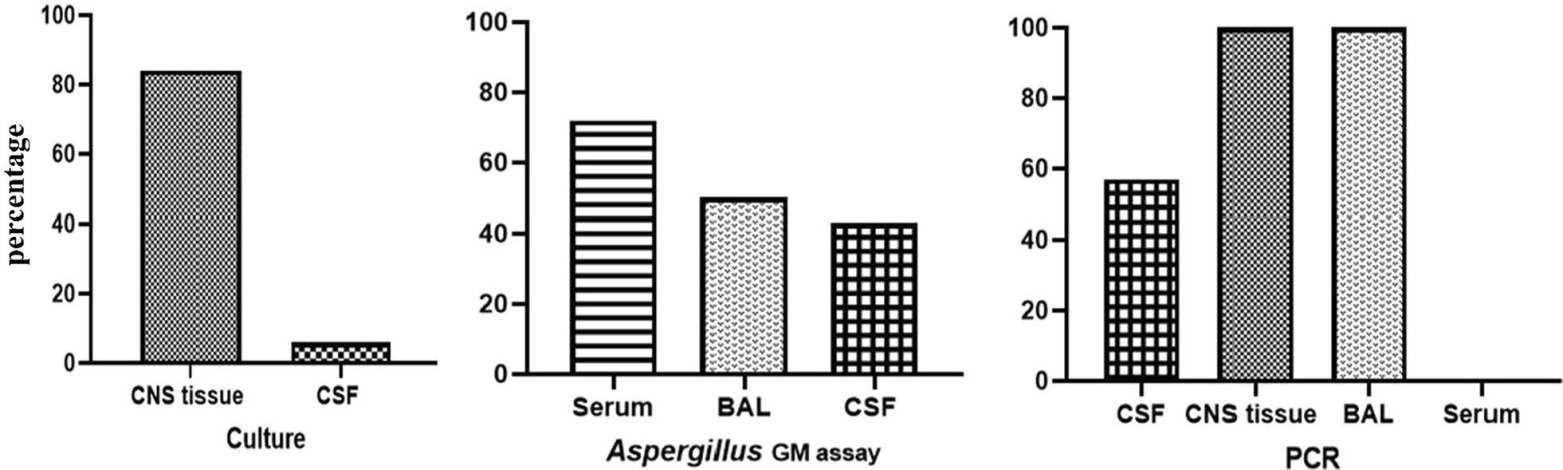
Positive results obtained from various samples for the diagnosis of CNS aspergillosis in patients with leukemia and stem cell transplantation (PubMed reported cases until August 2020). *CNS* Central nervous system, *CSF* Cerebrospinal fluid, *BAL* Bronchoalveolar lavage, *PCR* Polymerase chain reaction

Positive results of CSF *Aspergillus* GM assay and PCR led to the diagnosis of infection in a patient, while *Aspergillus* was not isolated in the CSF [20]. On the other hand, in another case of a 15-year-old girl undergoing autologous SCT, parents were reluctant to agree to proceeding with brain biopsy and serologic markers of fungal infection were negative. In this situation, fungal DNA was detected in the CSF by panfungal PCR assay using the primers derived from fungal 18S ribosomal RNA (rRNA) genes [21]. Of note, only 11 (18.6%) patients were diagnosed with PCR, and CSF (four (57%) positive, three negative), tissue (two positive), BAL (one positive), and serum (two negative) samples were used for diagnosis (Fig. 3). It is noteworthy to mention that CSF examination (cell count, glucose level, and protein content) was carried out in 19 cases, the result of which was normal in ten patients (52.6%).

Medical imaging modalities including Computed Tomography (CT) and Magnetic Resonance Imaging (MRI) did not yield a definitive diagnosis for any of the patients. However, among the patients with imaging modalities, abscesses appearing as ring-enhancing lesions (post-infarct abscess formation) (57.6%), perifocal edema on MRI scan (marked T2-hyperintense peripheral edema and marked T1-hypointense peripheral edema) (56%), brain hemorrhages (3.3%), and nonspecific hyper intense foci (1.7%) were the most common signs reported. In the case of a Japanese patient with AML and *Aspergillus* meningitis, CT of the brain was unremarkable; however, MRI scans showed abnormal meningeal enhancement [20].

In 12 other patients (20.4%), pulmonary aspergillosis was diagnosed using BAL, bronchial washing and sputum culture, and lung biopsy. In this regard, CNS aspergillosis was diagnosed only after pulmonary aspergillosis and observations of lesions in the CNS, and no specific diagnostic method was used to confirm CNS aspergillosis. Finally, thirty-six cases (61%) of CNS aspergillosis were presented with additional non-CNS sites of invasive aspergillosis. The affected sites among these cases were the lungs (34 cases), bone, kidneys, heart and thyroid gland (two each), eyes, sinus, liver, and intramuscular and subcutaneous abscesses (one each) (Additional file 1: Table S1).

### Treatment

Various antifungal drugs have been used to treat CNS aspergillosis. The prevalent use of drug for treating this infection is amphotericin B (AMB) (76%), either deoxycholate (DAMB) or liposomal AMB. In five patients, the use of DAMB showed a good therapeutic function. DAMB was combined with itraconazole and voriconazole (two cases) and was administered locally in the surgical cavity for two other patients. The combined use of liposomal AMB with voriconazole and isavuconazole (every two cases) controlled infection in patients (Table 1). Notably, the AMB-resistant infection was reported in two patients. One of these patients had a DAMB-resistant pulmonary and cerebral aspergillosis (during treatment with humanized monoclonal antibody anti CD52), which was treated with voriconazole. Another patient in a case suffered a fatal CNS aspergillosis caused by *A. terreus*, an amphotericin-resistant mold, and he was mistakenly treated by empirical antifungal therapy including intrathecal AMB and then, died as a result [11, 22].

Voriconazole was another drug administered to a large number of patients (64%). According to our analysis results, voriconazole has been used to treat CNS aspergillosis since 2003, whereas before this year, it was used only for a patient in a phase-II clinical trial. In this patient, DAMB was changed to LAMB due to renal toxicity; then, antifungal therapy was continued with itraconazole; however, due to the development of the paraventricular lesion and an additional lesion in the cerebellum, the patients were subsequently enrolled in a phase-II trial and then, received voriconazole [23]. On the other hand, voriconazole has not been administered to 11 patients after 2003. In one of these patients, voriconazole could not be administered because of the hyperbilirubinemia; instead, DAMB was used, which did not affect the treatment process and the patient died as a result [14] (the antifungal drugs used for these patients are listed in Table 1). Side effects of voriconazole were reported in nine patients (23%) such as severe cytolytic hepatitis, transient visual disturbances, reversible elevation of the alkaline phosphatase, respiratory insufficiency, gastrointestinal disturbances, significant photosensitivity, and nail changes. It should be noted that, voriconazole monitoring was performed in 14 (36.8%) patients and the duration of using this drug by patients was reported in 16 cases with an average of 10.7 months.

Among the patients that were treated by voriconazole, the antifungal agent did not exhibit proper therapeutic function effects in 12 patients (31.5%) [3, 4, 10, 22, 24–31]. In four of these patients, the antifungal agent was replaced with other drugs due to its possible side effects [10, 24–26]. Besides, in another patient, voriconazole was replaced by isavuconazole due to coinfection with Mucorales [32]. Voriconazole in 58.3% of these patients was not monitored. Finally, the combined use of voriconazole and liposomal AMB (three cases), caspofungin (there cases), DAMB (two cases), and micafungin led to controlling CNS aspergillosis [2, 33–40]. In addition, in a patient with CLL, signs of intracranial hyper-tension with generalized seizure were developed despite taking voriconazole. Prednisone (40 mg/day) was added to this antifungal agent which led to the gradual improvement of the patient’s condition [37]. In addition to AMB and voriconazole, other antifungals such as caspofungin (11 cases), itraconazole (nine cases), isavuconazole (five cases), flucytosine and micafungin (four each), fluconazole, and posaconazole (three each) were used (Table 2). Notably, prophylactic and empirical antifungal agents were used for treatment to control fungal infections in patients with leukemia or SCT. However, in almost all of the cases, no improvement was achieved (Additional file 1: Table S2).

**Table 2:** Confirmation methods and treatment of CNS aspergillosis in patients with leukemia or stem cell transplantations

Confirmation methods	
Histopathological examination (HE)	39%
HE and culture	18.70%
Culture	5%
Post-mortem	5%
*Aspergillus* galactomannan (GM)	8.50%
GM assay and PCR	1.70%
PCR	1.70%
Non-CNS samples	20.40%
Medical imaging modalities	
Ring enhancing lesions	57%
Perifocal edema on MRI	56%
Brain hemorrhages	3.30%
Nonspecific hyper intense foci	1.70%
Treatment	
Amphotericin B	76%
Voriconazole	64%
Caspofungin	18.60%
Itraconazole	15.20%
Isavuconazole	8.50%
Flucytosine	6.70%
Micafungin	6.70%
Fluconazole	5%
Posaconazole	5%
Surgery	39%

Besides antifungal therapy, surgical intervention appears to have a key role in the treatment of CNS aspergillosis. Surgery was used in 23 (39%) patients. These surgeries included Craniotomy with aspiration and resection of brain abscesses, stereotactic resection, aspiration, and drainage. Besides, one female patient with ALL underwent T11–L1 laminectomy and ultrasound-guided aspiration for her intramedullary and extra medullary abscesses [28]. The main surgical finding was a vascularized thick capsule (soft capsule) containing a necrotic purulent component, pinkish white pus or viscous fluid [25, 41, 42]. In one patient, after five weeks of DAMB use, the patient’s symptoms worsened; therefore, resection of the inferior temporal lobe abscesses and debridement of the external canal, petrous apex, and mastoid air cells were performed, which led to control of the infection and recovery of the patient [5]. On the other hand, a patient with SCT underwent total surgical removal of the cerebellar abscess with suboccipital craniectomy; however, after 5 weeks, the scan again revealed an intra-cerebellar abscess. In this condition, the patient underwent reoperation and was treated with locally DAMB and itraconazole [43]. Stereotactic or open surgery were recommended in five patients, which was not possible due to severe thrombocytopenia and underlying hematological conditions, critical localization of the lesion, in light of the formation of abscess capsule and the patient’s general condition [2, 3, 18, 31, 33]. In another patient, the neurosurgical procedure was not considered due to the risk that the organism would penetrate the brain; however, the patient successfully recovered with catheter coil embolization and long-term antifungal agents [44].

## Discussion

*Aspergillus* species are common contaminants of the upper respiratory tract with initial colonization occurring in the nasopharynx or lower respiratory tree. However, in patients with leukemia or prolonged neutropenia, hematogenous dissemination from the lung and secondary cerebral aspergillosis cause a significant mortality rate [6, 7]. Recent study reported acute leukemia as the most common underlying disease in patients with fungal infections of the CNS and paranasal sinuses [62] and our results also showed ALL as the most common leukemia in patients. In the present study, the overall mortality was 34%; however, the mortality attributed to CNS aspergillosis was 24.5%. On the other hand, in a systematic review of reported cases (ninety cases recorded up to June 2005) on CNS aspergillosis in children, as published by Dotis et al., the overall mortality rate was 65.4% [63]. Such a high mortality rate can be related to the screening of the disease in infants and children, the unavailability of antifungal such as voriconazole, and the screening of patients with all the underlying disorders. In this regard, two recent studies have reported 33% and 48% mortality rates for invasive fungal infections of the CNS. They suggested that the mortality remained high; however, compared to previous historical data, it seemed to have been reduced, probably due to the availability of newer antifungal drugs, immune response in histopathology, absence of co-infections, corticosteroid tapering, and possibly surgical drainage [62, 64].

From the data available in the literature, altered mental status, hemiparesis, cranial nerve palsies, and seizures were the clearest manifestations of CNS aspergillosis. Besides, our results showed lung involvement in more than half of the patients and 61% of the cases presented with additional non-CNS sites of invasive aspergillosis. These results should motivate clinicians to rule out CNS aspergillosis quickly and efficiently in patients suffering pulmonary aspergillosis [41]. In this regard, in a patient with AML, before the initiation of induction chemotherapy, MRI of the brain was conducted and it did not detect any intraparenchymal brain abnormalities. However, 16 days after induction chemotherapy, the patient developed pulmonary symptoms and three days later, brain involvement occurred [22]. Therefore, the onset of CSN aspergillosis in patients with leukemia and immunodeficiency is very rapid, which requires greater control and following up of patients. Ibrutinib has been used in recent years to treat CLL patients. Our results showed that, CNS aspergillosis in patients occurs 6 months, on average, after ibrutinib use, suggesting that CNS is a safe haven for invasive aspergillosis in all CLL-induced patients treated with ibrutinib. In this respect, meticulous and repeated neurological examinations and fast diagnosis are needed for patients with invasive aspergillosis after ibrutinib treatment, with a very low threshold for prescribing MRI of the brain [37].

Given that the clinical signs of CNS aspergillosis are usually nonspecific and similar to other diseases, differential diagnosis such as lung cancer, cerebral infection or abscesses such as listeriosis, cryptococcal and tuberculous meningitis, metastatic disease and cerebral malignancy should be considered when imaging modalities are used for diagnosis in patients [39]. Noteworthy, the MRI appearance of CNS aspergillosis depends on different factors such as the timing of neuroradiologic assessment, immunologic status of the patient, and the characteristics of the fungus [42]. However, our results showed that on conventional MRI sequences in patients with leukemia, CNS aspergillosis appears as ring-enhancing lesions, with perifocal edema on MRI. Brain CT has not proven useful in the case of *Aspergillus* meningitis, which has no parenchymal lesions, while Gadolinium-enhanced MRI of the brain ensures a more efficient diagnosis of the infection [20, 65]. As mentioned earlier, CSF analysis was normal in most of the patients because neutropenic patients with fungal meningitis do not always show elevation of the CSF cell count [20]. Therefore, imaging modalities are subject to many limitations for accurate diagnosis of CNS aspergillosis; but, if CT and MRI are indicative of cerebral lesions and infarction and vascular inflammation in an immune-compromised host, a fungal etiology must be considered, even if CSF examination does not reveal any abnormalities [7].

Histopathological examination and the use of brain biopsy have been the most commonly used diagnostic methods for patients; however, given that a large number of patients are children, parents are often reluctant to proceed with brain biopsy [21]. In addition, due to coagulation issues and underlying hematological conditions, applying an invasive diagnostic procedure is not always feasible for patients with leukemia [3]. In this regard, a study reported that performing MR-guided biopsy of the suspected brain lesion can yield a more precise tissue diagnosis and its feasibility is proven for sick leukemia patients during remission induction and it allows for intra-lesional local instillation of drugs required [10]. On the other hand, even with a proper biopsy, histopathological examinations may not show the diagnostic features of fungal infections [45]. Besides, it is quite challenging to make a diagnosis of CNS aspergillosis on a histomorphological basis and the most prevalent cause for incorrect morphological diagnosis is the misidentification of *Mucorales* as *Aspergillus* spp [66, 67]. Due to mixed mold infection and antifungal resistance, identification of *Aspergillus* at the species level should be considered in multiple site involvement [4]. Therefore, histopathological examination of different samples has limitations for diagnosis and for some patients, other ways such as culture and molecular methods should be used for the species-level identification and definitive diagnosis of infection.

Culture was used to diagnose CNS aspergillosis in 23% of the patients. It should be mentioned that in some patients, prolonging the culture time of the microorganism reduces the diagnostic value of this method. For example, in a patient with APL, the histologic evaluation proved diagnostic for aspergillosis, while the cultures became positive only 3 weeks later [5]. In addition, even after identifying a mold on the culture media, it still requires several more days to detect the fungus at the species level [22]. Our results showed that when samples obtained from biopsy or surgery were used for culture, there was a higher chance of isolating *Aspergillus*, while the use of CSF was not very desirable for culture. In this context, as mentioned, obtaining tissue samples in patients with leukemia is highly restricted. Therefore, because early identification of opportunistic invasive fungal pathogens has been shown to guide interventions and affect prognosis, culture may be limited in patients with challenging conditions.

In this regard, the use of molecular methods for diagnosing the cause of infection and drug resistance can be helpful. The use of PCR should be considered in two situations: (A) when the levels of fungi in both blood and CSF are below the lower limit of detection by conventional diagnostic assays; (B) when an uncommon fungal pathogen, which remains undetected by conventional diagnostic assays, infects the CNS [21]. In a patient with AML, *A. terreus* was detected using PCR and Electrospray Ionization with Mass Spectrometry. This pathogen is inherently resistant to AMB and rapid diagnosis can prevent therapeutic fractures in patients [22]. Therefore, timely identification of CNS aspergillosis by molecular methods can lead to the institution of pathogen-specific and directed therapy and should be used more in patients.

*Aspergillus* GM assay was another diagnostic method for diagnosing infection in 8.4% of the patients. The GM test is an enzyme-based immunological method used to determine the GM exo-antigen of *Aspergillus* species in the cell wall [35]. Recent studies have reported low sensitivity to PCR, considering that only a small number of fungal cells are observed in the CSF. Alternatively, GM assay in the CSF was considered to be the most useful [19, 65, 68]. However, our results showed the superiority of PCR in detecting CNS aspergillosis from CSF samples. Moreover, present study demonstrated that serum and BAL samples were more suitable for performing GM assay than CSF. Notably, GM assay showed cross-reactivity with other hyalohyphomycetes such as *Fusarium* [69, 70]. However, when culture and PCR of CSF were negative in one patient, GM assay alone led to a correct diagnosis of the infection. Furthermore, decline of the GM antigen titer during treatment corresponded to the clinical response to treatment [19, 65]. More importantly, accurate diagnosis using GM assay demands multiple sampling and serial *Aspergillus* GM monitoring is useful in the early detection of relapse and reinitiation of antifungal therapy [27]. Thus, as mentioned before, each of the diagnostic methods of CNS aspergillosis in patients with blood malignancies has advantages and limitations. Therefore, if possible, using the most appropriate sample for each test can increase the chances of detecting a fungal infection. Clinicians should use diagnostic methods according to the patients’ condition to ensure correct diagnosis of the infection.

After proper and timely diagnosis, the use of appropriate antifungal drugs is also very important. AMB and voriconazole are the most commonly used antifungals in patients with CNS aspergillosis. AMB, the echinocandins, itraconazole, and posaconazole are large molecules and the penetration of these drugs across the blood–brain barrier is mainly limited. Fluconazole and 5-fluorocytosine penetrate well into the CNS; however, *Aspergillus* frequently exhibits resistance to these antifungal agents [71]. Voriconazole displays a broad range of antifungal activities and facilitates CNS penetration. The 2017 ESCMID guidelines recommend voriconazole as the first-line agent for “proven” or “probable” aspergillosis treatment in all children [72]. However, our analysis showed that good results were not obtained in one-third of patients treated with voriconazole.

Therapeutic Drug Monitoring (TDM) is highly recommended when voriconazole is used, because achieving therapeutic concentrations in a timely manner can be challenging due to nonlinear pharmacokinetics and observed inter-patient variability. In this regard, it is still difficult to find the most effective, yet tolerated, dose, primarily due to the poor correlation between dose and serum concentration. Our results showed that TDM of voriconazole has not been performed for a range of patients which could be due to limited access to the serum voriconazole level testing and slow turnaround time. Most significantly, individuals with sub-therapeutic concentrations are at increased risk of mortality. On the other hand, high voriconazole concentrations may cause adverse effects like neurotoxicity and hepatotoxicity [3, 61, 73]. Furthermore, clinicians should be cognizant of the drug-drug interaction between voriconazole and corticosteroids for cytochrome P450 isoenzymes, CY3A4, CYP2C9, and CYP2C19, which can lead to decreased plasma voriconazole concentrations and, thus, limited efficacy against the *Aspergillus* [10, 31]. Therefore, to prevent voriconazole therapy failure, it is imperative to attain therapeutic voriconazole plasma concentrations promptly in order to achieve a favorable response and also, is necessary to perform a CYP2C19 genotype test to determine the genetically predicted metabolizer status can prevent therapeutic failures when voriconazole is used.

In some patients, the use of combination therapy showed good performance. However, characterization of patients benefiting from a combination antifungal therapy is required and confirmatory results of further prospective studies are needed before the combination therapy of antifungal agents can be fully accepted as standard strategies for CNS aspergillosis. Lastly, there are no clear recommendations as to the exact duration of antifungal treatment of mold infections of the CNS. However, antifungal chemotherapy is usually recommended until the resolution of all clinical, laboratory, and radiographic findings of active infection [52]. Besides, following the treatment of CNS aspergillosis in leukemia patients, prolonged and, in some cases, lifelong secondary prophylaxis may be necessary after the initial treatment [27].

Neurosurgical intervention was used in 39% of the patients. One study reported that the use of image-guided stereotactic neurosurgery provided a safe and vital component in the successful treatment of patients’ devastating conditions [41]. In another patient with bone marrow transplantation, despite the administration of AMB, flucytosine, and micafungin, the patient died 2 months after transplantation. The authors suggested that if the infected lesion remains after antifungal agent’s therapy, surgical drainage or resection of infected tissue along with systemic therapy may be important. In this regard, Infectious Diseases Society of America (IDSA) guidelines recommend surgical drainage and infected tissue removal along with systemic antifungal therapy for patients suffering from CNS aspergillosis [52]. Therefore, using a combination of antifungals along with surgery can help control the infection. In some cases, due to the critical localizations of the lesion and underlying hematological conditions, surgery is not possible. In this situation, the use of antifungal agents continues for a very long time [2].

Finally, in addition to surgical intervention and antifungal treatment, patient induction chemotherapy management, parallel resolution of neutropenia, and complete remission of leukemia undoubtedly play an important role in treating patients. A female patient with CLL was treated with voriconazole after being diagnosed with CNS aspergillosis. Then, she received bendamustin for CLL progression, leading to more profound neutropenia and clinical deterioration [26]. Therefore, along with the mentioned treatments, it is important to control the patient’s underlying conditions, which can facilitate the treatment process. For instance, discontinuing immunosuppressive drugs, if possible, can help control CNS aspergillosis.

## Conclusion

CNS aspergillosis is a highly lethal disease in patients with blood malignancies and is subject to a very poor prognosis. Patients with leukemia are very sensitive to fungal infections due to underlying disorders and several previous chemotherapy regimens. In this regard, pulmonary involvement in these patients usually occurs shortly after the start of chemotherapy and after that, CNS infections may occur as an occult asymptomatic extra-pulmonary involvement during the diagnostic evaluation of febrile neutropenic patients or symptomatic form, which usually develop after a few weeks of pulmonary manifestation. Therefore, systematic full screening including CT scan and enhanced MRI for CNS lesions should be performed for every diagnosis of invasive fungal infection; in addition, when infection is suspected in these patients, a definitive and differential diagnosis should be made using various diagnostic methods. If possible, species identification of the fungus is suggested because the occurrence of antibiotic resistance in some species can completely change the treatment regimen. In addition, the use of combination therapies should be considered in future studies so that if the first line of the treatment fails, the most appropriate treatment strategy can be adopted for patients. Furthermore, in addition to using appropriate antifungal therapy and TDM, control of patients’ chemotherapy should also be considered because the outcome of invasive aspergillosis is poor unless immunologic status improves.

## Supplementary Information

**Additional file 1: Table S1.** Various findings that led to the diagnosis of CNS aspergillosis in patients with leukemia or stem cell transplantation. **Table S2.** Prophylaxis and empirical antibiotic therapy for patients with inducing chemotherapy or stem cell transplantation. **Table S3.** *Aspergillus* Galactomannan assay in patients with CNS aspergillosis and leukemia or stem cell transplantation.

## Data Availability

The authors confirm that the data supporting the findings of this study is available within the article and its supplementary materials.
