# Supplementary material for: Aspergillosis of central nervous system in patients with leukemia and stem cell transplantation: a systematic review of case reports
Source: Ann Clin Microbiol Antimicrob. 2021 Jun 15;20:44. doi: 10.1186/s12941-021-00452-9 (PMC8204492; doi:10.1186/s12941-021-00452-9)
Supplement: Supplementary file 1 — Additional file 1: Table S1. Various findings that led to the diagnosis of CNS aspergillosis in patients with leukemia or stem cell transplantation. Table S2. Prophylaxis and empirical antibiotic therapy for patients with inducing chemotherapy or stem cell transplantation. Table S3. Aspergillus Galactomannan assay in patients with CNS aspergillosis and leukemia or stem cell transplantation. [file 12941_2021_452_MOESM1_ESM.docx]

**Table S1:** Various findings that led to the diagnosis of CNS aspergillosis in patients with leukemia or stem cell transplantation (PubMed reported cases until August 2020).

| **Country, year of publication and references** | **Histopathologic examinations** | **Computed tomography** | **Magnetic resonance imaging** | **Chest x ray or CT** | **Additional non-CNS sites of invasive aspergillosis** |
| --- | --- | --- | --- | --- | --- |
| Australia, 1995  [45] | Specimen showed necrotic brain substance with invasion by polymorphs and fibrin. Blood vessels were cuffed by lymphocytes and occasional polymorphs | several right-sided frontal lesions with ring enhancement after administration of contrast material. There was no evidence of edema or mass effect | NR | Nothing abnormal | NR |
| Australia, 2019  [25] | Gliosis and filamentous septate hyphae with acute angle branching | NR | Multiple rim enhancing hemorrhagic lesions, the largest of which was located in the left parieto-temporal region | A 5mm pulmonary nodule | Lung |
| Austria, 1999  [46] | Mycotic hyphae consistent with Aspergillus fumigatus. | A subdural hematoma of the left parietal region. | An aneurysm of the left arteria cerebri media, 6 mm in diameter, and no signs of cerebral aspergillosis. | A diffuse infiltrate in the right upper lobe of the lung. | Bone |
| Belgium, 1999  [7] | Parenchymal necrosis with a few lymphocytes and granulocytes and filaments of aspergillus. | NR | Small lesions consistent with acute infarcts in the temporal lobe and in the right lentiform nucleus seen as hyper intense on proton-density and T2-weighted sequences. | NR | Lung, kidney and liver |
| China, 2015  [18] | In light of the formation of abscess capsule, transcranial puncture was not performed | Hypodense lesions in his occipital and parietal lobes. | High signal intensity areas surrounded by a peripheral rim of hypodense signal with accompanying cerebral edema | Multiple nodular lesions and patchy floccular shadows were demonstrated | Lung |
| Czech Republic, 2005  [10] | Septated hyphae with sharp angles, consistent with Aspergillus spp. | A space-occupying lesion in her frontal lobes, crossing the midline, 44 × 36 × 38mm,reaching lateral ventricles, with post contrast ring enhancement | A space-occupying lesion in her frontal lobes, crossing the midline, 44 × 36 × 38mm,reaching lateral ventricles, with post contrast ring enhancement | NR | NR |
| France  [37] | Diagnosis of abscesses with septate hyphae compatible with *Aspergillus* sp. | NR | Revealed multiple nodular lesions | Left pleural effusion and bilateral nodules | Lung |
| France, 2001  [47] | NR | NR | Multiple fraction | Pulmonary invasive aspergillosis | Lung |
| France, 2003  [33] | It was not done due to the patient's bad condition. | Five nodular lesions with perifocal edema in the frontal and parietal lobes. | NR | Pulmonary infiltrates | Lung |
| France, 2018  [37] | NR | NR | Several abscesses | Bilateral nodules and enlarged mediastinal lymph nodes. | Lung |
| France, 2019  [26] | Acute septate thin hyaline filamentous fungi | NR | Several cerebral abscesses | NR | NR |
| France, 2019  [48] | NR | NR | Nodular lesions consistent with cerebral IA associated with brain edema | Bilateral upper lobe lung nodules; | Lung |
| France, 2019  [32] | NR | A well-defined rim-enhancing lesion with a hypodense center surrounded by oedema in the left external capsule region complicated by a mass effect on the left ventricle and a subfalcine herniation | NR | NR | NR |
| France, 2020  [31] | It was not done due to the sever neutropenia. | NR | Multiple abscesses consistent with cerebral aspergillosis | Bilateral upper lobe lung nodules | Lung |
| Germany, 1997  [49] | Aspergillosis | NR | Two parieto-occipital abscess formations were detected measuring 3.5 cm (left hemisphere) and 2 cm (right hemisphere) in diameter. Lesions revealed an irregular ring of contrast enhancement typical for aspergillus brain abscess | Presence of a left subhilar pulmonary aspergilloma measuring 4 cm in diameter | Lung |
| Germany, 1997  [23] | Contained septate hyphae typical for the Aspergillus species. | NR | A ring enhancing brain abscess with distortion of the left lateral ventricle | NR | Lung |
| Germany, 2017  [24] | *A. fumigatus* | NR | Least 4 intracerebral abscesses and signs of increased intracranial pressure | Diffuse, fine-nodular pulmonary transparency-reduction with a marked opacification of the right lower lobe. infection. | Lung |
| Germany, 2017  [50] | necrotizing granulomatous inflammation with detection of fungal mycelium, most likely Aspergillus species | NR | Multiple cerebral abscesses | Atypical pneumonia with possible fungal involvement | Lung |
| Greece, 2013  [51] | Hyphae with branches of 45 degrees were seen | NR | Right frontal lesion with surrounding edema, which was ring enhancing after contrast administration | NR | NR |
| India, 2012  [14] | Vasoinvasive aggregates of fungal hyphal forms with septations and branching suggestive of aspergillosis | Multiple well defined, enhancing, hypodense nodules in both cerebral hemispheres with perilesional edema. | NR | Bilateral multiple nodules surrounded by halo, suggestive of angioinvasive pulmonary aspergillosis | Lung |
| Iran, 2020  [4] | Septate hyphae | Extensive left hemisphere intracerebral hemorrhage in the first brain CT scan and multiple varying size ring like enhancing lesions within the different parts of white matter of both cerebral and cerebellar hemispheres representing abscess formation | Multiple brain abscesses | Small ground-glass opacities in the right and left hemi thorax | Lung |
| Italy, 2003  [11] | NR | A cranial CT scan without contrast in the first 24 hours was negative. | A right frontal-parietal lesion, 2 x 1,5 cm in diameter, with ring enhancement and perilesional oedema | Pulmonary infiltrate in the median field, 3 x 4 cm in diameter with halo sign | Lung |
| Italy, 2011  [42] | *Aspergillus fumigatus* | A small upper left cerebellar cortical-subcortical hypodense area close to a round ring-like hyper dense lesion without surrounding edema. | A multi lobular extra-axial mass lesion arising both from the inferior surface and from the free margin of the cerebellar tentorium. T2-weighed images revealed mild surrounding edema and a thin, irregular peripheral hypointense ring. | Normal | NR |
| Italy, 2018  [40] | Necrotizing encephalitis with mycelial hyphae compatible with Aspergillus. | A 10-mm contrast-enhancing cerebral mass in the left parietal lobe and smaller similar lesions in the cerebellum and right hemisphere. | NR | Pulmonary nodules with cavitation | Lung |
| Italy, 2019  [3] | It was not done due to the hematological condition. | NR | A left frontal hypointense lesion with peripheral contrast enhancement after gadolinium injection, suggestive of brain abscess in T2-weighted and diffusion weighted images | A characteristic fungal ball with the air crescent sign in the left upper lobe. | Lung |
| Japan, 2004  [21] | NR | NR | Ring-enhancing lesions in both basal ganglia. | Normal | NR |
| Japan, 2007  [20] | NR | Normal | Abnormal meningeal enhancement | Normal | NR |
| Japan, 2008  [52] | Aspergillosis | NR | A cerebral abscess in the left occipital lobe. | Interstitial shadowing in the bilateral lungs | Lung, kidney, heart |
| Japan, 2008  [44] | Necrotic debris and numerous fungal organisms with separate hyphae and dichotomous branching, consistent with Aspergillus species | NR | A large left-lobe lesion, which was compatible with hemorrhagic infarction and aspergillotic lesion in the temporal bone | NR | NR |
| Japan, 2020  [19] | NR | Low-density area on the left lenticular nucleus | An infarction of the left internal striatum artery | NR | NR |
| Japan, 2020  [29] | NR | NR | Multiple high-intensity areas in the images of fluid-attenuated inversion recovery (FLAIR), suggesting posterior reversible encephalopathy syndrome or multiple cerebral infarctions | Pneumonitis | Lung |
| Netherlands, 2008  [34] | NR | Multiple intracerebral hypodense lesions. | NR | Multiple lesions throughout both lungs, some surrounded by ground glass aspect, suggestive of pulmonary aspergillosis. | Lung, thyroid gland |
| Portugal, 2005  [12] | Aspergillosis | A large left parietal lobe lesion. | Low intensity in T1 and a ring enhanced by contrast. In T2, the lesion had increased signal, a central necrotic area and a low intensity ring. There was surrounding oedema and lateral ventricle compression, with no evidence of obstruction. | NR | NR |
| Spain, 1997  [53] | Aspergillosis | Multiple hypodense lesions in both cerebral hemispheres that showed no enhancement after the addition of intravenous contrast. | NR | Alveolar infiltrates in the lower lobes of both lungs. | Lung, thyroid gland |
| Sweden, 2012  [54] | NR | Slight progression with the perifocal oedema. | Four abscesses with peripheral contrast enhancement and slight progression of perifocal oedema | Multiple nodules, partially confluent with lesions in both lungs | Lung, sinus |
| Turkey, 1997  [55] | Fungi with septate and angulating hyphae | A round lesion in the right frontal lobe. | NR | Infiltrate in the lower segment of the upper right lobe | Lung |
| Turkey, 2002  [43] | Branching septate hyphae characteristic of Aspergillus were visible on hematoxin and eosin-stained slides. GMS stain allowed the organisms to be visualized more clearly | A large right cerebellar lesion, with ring like contrast enhancement. | A large right cerebellar lesion, with ring like contrast enhancement. | NR | NR |
| Turkey, 2012  [2] | Extensive necrosis, acute inflammation, and septate fungal hyphae branching with narrow angle. | NR | Nodular lesions located at the right frontal lobe, left cerebellum, and cingulate gyrus. The nodules all show complete ring-like peripheral enhancement surrounded by perilesional vasogenic edema | Normal | NR |
| Turkey, 2013  [35] | 45-degree branching dichotomous hyphae and large septate hyphae | NR | A lesion of 12 mm size in the right frontal lobe and nodular lesions on the left cerebellum and on the cingulate gyrus in the superior part of the left corpus callosum were detected. | NR | NR |
| Turkey, 2018  [56] | Inside the necrotic areas, abundant fungal organisms proliferating in the walls and lumens of vascular structures were observed. GMS stain revealed that fungal organisms form uniform, slender, acutely branching, septate | NR | Multiple lesions in the brain stem, bilateral occipital and right frontal lobes | NR | NR |
| Turkey, 2018  [56] | Aspergillosis | NR | There was a lesion at the right frontal lobe, approximately 55x60 mm in size with a peripheral edema and peripheral contrast enhancement | NR | NR |
| Turkey, 2018  [56] | Aspergillosis | NR | Cerebellar lesion was seen on MRI | NR | NR |
| UK, 2000  [57] | Aspergillus fumigatus hyphae. | NR | A solitary lesion of high-density signal in the right parietal cortex, which enhanced with gadolinium on T1 image. The mass was 3 cm in diameter and surrounded by vasogenic edema | Patchy changes | NR |
| UK, 2006  [58] | NR | Ring enhancing lesion in right cerebral hemisphere highly suggestive of aspergillosis. | NR | Right mid zone air-crescent sign highly suggestive of pulmonary aspergillosis | Lung |
| UK, 2015  [36] | The presence of fungal hyphae | NR | 4 cerebral parenchymal lesions, with evidence of irregular cavitation, central enhancement, and surrounding edema |  | NR |
| USA, 1991  [5] | Typical large septated dichotomous hyphae with regular angular branching were identified. | Left temporal cerebritis and destruction of the left petrous bone, mastoid air cells, and external auditory canal complex. More superiorly, the temporal lobe appears diffusely hypodense around well-circumscribed ring-enhancing daughter lesions | Left temporal cerebritis and destruction of the left petrous bone, mastoid air cells, and external auditory canal complex. | Right lower lobe pneumonic infiltrate. | Lung |
| USA, 1998  [6] | Septate hyphae consistent with aspergillosis | NR | Multiple-enhancing lesions in the right parietal, right temporal, left midbrain, and left thalamus and mild ventriculomegaly consistent with fungal lesions | NR | NR |
| USA, 2005  [41] | Aspergillosis | A round, hyper dense mass in the right frontal lobe. | NR | A pulmonary lesion | Lung |
| USA, 2005  [41] | Aspergillosis | NR | Revealed three hyper intense lesions, one left parietal lobe lesion and two right-sided temporoparietal lesions | NR | Lung |
| USA, 2005  [41] | Aspergillosis | Two intra axial lesions. | NR | NR | NR |
| USA, 2012  [22] | Many septate hyphae with dichotomous branching within fungal balls. Aspergillus species | NR | Eight lesions in the brain associated with faint incomplete contrast enhancement | A left lingular infiltrate consistent with either atelectasis or pneumonia | Lung |
| USA, 2014  [27] | Angioinvasive branching septated fungal hyphae with uniform diameter in brain tissue, which was suggestive of Aspergillus spp | NR | Multiple bilateral infarcts and diffuse ring-enhancing lesions involving both hemispheres of the brain. | Focal opacities in left middle ,left upper, and right lower lung and possible reverse halo sign | Lung, bone |
| USA, 2014  [59] | NR | Air emboli | Multiple areas of restricted diffusion within the left MCA territory | A diffuse severe pneumonia | Lung |
| USA, 2014  [28] | Intraoperative specimen showed abundant hyphae | NR | Rim-enhancing, expansile intramedullary lesion within the distal spinal cord at T12–L1, most consistent with abscess | NR | NR |
| USA, 2018  [38] | Septate filamentous fungi with acute angle branching characteristic of Aspergillus fumigatus | NR | Rim-enhancing lesions with diffusion restriction in the right peritrigonal and left corona radiata | NR | Eye |
| USA, 2018  [39] | NR | Multiple small hypodense lesions with associated edema. | Multiple ring-enhancing lesions in right cerebellar peduncle, cerebellar vermis left occipital, right temporoparietal, bilateral frontal, and a large lesion in the right parietal cortex near the vertex | Consolidation in left upper lobe and worsening generalized lymphadenopathy | Lung |
| USA, 2019  [60] | Necrosis, acute inflammation and granulation tissue consistent with an abscess and a GMS stain highlighting septate hyphae. | A large 3.2 cm (cm) hyper dense mass in the left cerebral hemisphere with surrounding vasogenic edema, a mass effect on the left lateral ventricle and small hyperdense masses in the right cerebral hemisphere. | A 3.2 cm round heterogeneous mass noted in the left parieto-temporal region, a 12 mm (mm) mass in the right occipital lobe and an oval shaped 8 mm mass in the right frontal lobe | A new spiculated lung nodule within the right lower lobe measuring 1.5 × 1.7 cm with surrounding ground glass opacity. | Lung |
| USA, 2019  [30] | Numerous fungal hyphae with dichotomous branching and associated tissue necrosis. | NR | Multifocal ring-enhancing lesions with centrally restricted diffusion throughout bilateral cerebral hemispheres and right cerebellum concerning for multifocal abscesses | Multiple pulmonary nodules, and left lung consolidation | Lung, heart,  intramuscular, and subcutaneous abscesses |
| USA, 2020  [61] | Acute 45-degree angle branching hyphae | NR | A ring enhancing lesion in the left occipital region without sinus abnormalities | NR | NR |

**Table S2:** Prophylaxis and mpirical antibiotic therapy for patients with inducing chemotherapy or stem cell transplantation (PubMed reported cases until August 2020).

| **Country, year and reference** | **Prophylaxis** | **Empirical treatment** | **Outcome** |
| --- | --- | --- | --- |
| Belgium, 1999  [7] | NR | AMB | Extension of the infarcted areas and abscesses |
| Czech Republic, 2005  [10] | NR | AMB 0.8 mg/kg/day due to fever | Barefaced, gross, frontal behavior, followed by  qualitative and quantitative changes in her consciousness. |
| France, 2003  [33] | NR | AMB 1 mg/kg per day due to pulmonary infiltrates | Two days thereafter, the patient became withdrawn and confused. |
| Germany, 1997  [49] | Fluconazole | AMB (50 mg/d) | Multiple brain abscesses |
| Germany, 1997  [23] | NR | AMB 1·1 mg/kg/d. and LAMB 2·1 mg/kg/d. due to pleural chest pain and a pulmonary infiltrate. Then itraconazole due to fungal brain abscess | Progression of the paraventricular lesion and an additional lesion in the cerebellum |
| India, 2012  [14] | NR | AMB 0.5 mg/kg | Multiple large nodular  shadows predominantly in the lower lobes |
| Iran, 2020  [4] | LAMB | Voriconazole due to fever | Without any benefits |
| Italy, 2003  [11] | Itraconazole (400 mg daily) | NR | The patient developed left hemiparesis. |
| Italy, 2011  [42] | Itraconazole 350 mg p.o./d, | NR | Without any benefits |
| Japan, 2004  [21] | NR | Fluconazole (400 mg/day) due to fever | The administration of high-dose fluconazole was discontinued on day 13 but the patient complained of headache and weakness in the left hand and leg on day 21 |
| Japan, 2008  [52] | NR | Fluconazole due to lung infection | Increasing pleural effusion was  noted on the chest radiograph, and hypoxemia progressed |
| Japan, 2008  [44] | Fluconazole | AMB (1mg/kg/day) due to complain of left ear pain, and left facial nerve palsy | Without any benefits |
| Japan, 2020  [19] | NR | LAMB IV 3 mg/kg/day and 5-flucytosine (5-FC) PO 100 mg/kg/day for possible cryptococcal meningitis then, fluconazole PO 8 mg/kg/day | Acute infarction on the corpus callosum |
| Portugal, 2005  [12] | Fluconazole | 14 day-course of 5 mg/kg LAMB due to persistent fever without a site of infection, | Confusion and a declining level of consciousness. |
| Spain, 1997  [53] | Fluconazole 100 mg/12h, p.o.) | AMB (1 mg/kg/day, iv) due to alveolar infiltrates in the lower lobes of both lungs | Multiple brain abscesses |
| Turkey, 1997  [55] | Fluconazole | LAMB | Without any benefits |
| UK, 2000  [57] | AMB and fluconazole both 5mg/kg/day for 20 days | NR | Brain abscess |
| UK, 2015  [36] | NR | LAMB, voriconazole due to cerebral parenchymal lesions | NR |
| USA, 1991  [5] | NR | AMB (45 mg/day) due to right lower lobe pneumonic infiltrate | Multiple brain abscesses |
| USA, 1998  [6] | AMB | NR | The patient developed an altered mental status |
| USA, 2005  [41] | NR | Fluconazole due to mouth sores however, treatment was changed to LAMB after suspicion of *Aspergillus* | The patient began to show neurological deficits. |
| USA, 2012  [22] | Fluconazole | Voriconazole | Respiratory and neurologic status worsened |
| USA, 2014  [27] | Itraconazole | NR | she developed acute, bilateral, and severe loss of vision. |
| USA, 2019  [30] | NR | Voriconazole | Without any benefits |

AMB: amphotericin B. LAMB: liposomal amphotericin b. NA: not applicable.

**Table S3:** *Aspergillus* Galactomannan assay in patients with CNS aspergillosis and leukemia or stem cell transplantation (PubMed reported cases until August 2020).

| **Country, year of publication and references** | **Serum** | **CSF** | **BAL** | **Index**  **(**ng/mL) |
| --- | --- | --- | --- | --- |
| Australia, 2019  [25] | Negative | NR | NR | NR |
| France,  [37] | Positive | Negative | NR | 0.8 |
| France, 2003  [33] | Positive | Negative | NR | NR |
| France, 2018  [37] | Positive | Negative | NR | 1.9 |
| France, 2019  [48] | Positive | NR | Positive | NR |
| France, 2019  [32] | Negative | NR | NR | NR |
| France, 2020  [31] | Positive | NR | Negative | NR |
| Germany, 2017  [24] | Positive | Positive | Negative | 1.1 |
| Germany, 2017  [50] | Negative | Negative | NR | NR |
| Greece, 2013  [51] | Positive | Positive | NR | NR |
| India, 2012  [14] | Positive | NR | NR | 3 |
| Iran, 2020  [4] | Positive | Positive | NR | NR |
| Italy, 2003  [11] | Negative | NR | NR | NR |
| Italy, 2018  [40] | NR | Negative | Positive | 1.3 |
| Italy, 2019  [3] | Positive | Positive | NR | NR |
| Japan, 2004  [21] | Negative | Negative | NR | NR |
| Japan, 2007  [20] | Negative | Positive | NR | 2.2 |
| Japan, 2020  [19] | Positive | Positive | NR | 5 |
| Japan, 2020  [29] | Positive | Positive | NR | 5 |
| Netherlands, 2008  [34] | Positive | Negative | NR | 5 |
| Sweden, 2012  [54] | Positive | NR | NR | 3.8 |
| Turkey, 2013  [35] | Positive | NR | NR | 3.3 |
| USA, 2012  [22] | NR | Negative | NR | NR |
| USA, 2014  [27] | Positive | NR | NR | NR |
| USA, 2014  [59] | Positive | NR | NR | NR |
| USA, 2014  [28] | Positive | NR | NR | 3.6 |
| USA, 2019  [60] | Negative | NR | NR | NR |
| USA, 2019  [30] | NR | Negative | NR | NR |

NR: not reported.
